# Radio-Immunotherapy-Induced Immunogenic Cancer Cells as Basis for Induction of Systemic Anti-Tumor Immune Responses – Pre-Clinical Evidence and Ongoing Clinical Applications

**DOI:** 10.3389/fimmu.2015.00505

**Published:** 2015-10-08

**Authors:** Anja Derer, Lisa Deloch, Yvonne Rubner, Rainer Fietkau, Benjamin Frey, Udo S. Gaipl

**Affiliations:** ^1^Department of Radiation Oncology, University Hospital Erlangen, Friedrich-Alexander-Universität Erlangen-Nürnberg, Erlangen, Germany

**Keywords:** radiotherapy, abscopal effect, immune therapy, checkpoint inhibitors, PD-L1, DAMP, EGFR, anti-tumor immunity

## Abstract

Radiotherapy (RT) primarily aims to locally destroy the tumor via the induction of DNA damage in the tumor cells. However, the so-called *abscopal*, namely systemic and immune–mediated, effects of RT move over more and more in the focus of scientists and clinicians since combinations of local irradiation with immune therapy have been demonstrated to induce anti-tumor immunity. We here summarize changes of the phenotype and microenvironment of tumor cells after exposure to irradiation, chemotherapeutic agents, and immune modulating agents rendering the tumor more immunogenic. The impact of therapy-modified tumor cells and damage-associated molecular patterns on local and systemic control of the primary tumor, recurrent tumors, and metastases will be outlined. Finally, clinical studies affirming the bench-side findings of interactions and synergies of radiation therapy and immunotherapy will be discussed. Focus is set on combination of radio(chemo)therapy (RCT) with immune checkpoint inhibitors, growth factor inhibitors, and chimeric antigen receptor T-cell therapy. Well-deliberated combination of RCT with selected immune therapies and growth factor inhibitors bear the great potential to further improve anti-cancer therapies.

Radiotherapy (RT) is an integral part of multimodal cancer treatments (1). Besides its local mode of action on tumor cell DNA, it can induce systemic and immune-mediated anti-tumor responses, especially in combination with additional immune activation (2). The current review focuses on induction of immunogenic cancer cell death by RT and on interactions of RT with selected immune therapies to induce a long-lasting, local, and systemic tumor control.

According to the WHO, cancer incidences are expected to increase by over 50% until 2020 (3). With this in mind, the clinical management of treatment modalities is a big challenge for scientists and clinicians alike. Consequently, improving the understanding of cellular and molecular processes occurring in the patients during therapies will help to optimize the design of clinical trials and ultimately that of patient treatment as well. Common therapy options comprise surgery, RT, chemotherapy (CT), immunotherapy (IT), targeted therapy, hyperthermia (HT), and hormonal therapy, all of which are either administered as a stand-alone therapy or in various combinations. Out of all these options, over 50% of all cancer patients receive RT (4).

## RT-Induced DNA Damage

The clear-cut aim of RT is the deposition of a maximal dose of ionizing radiation (IR) in the tumor while simultaneously sparing healthy tissue. A significant amount of damage within the malignant cells ultimately leads to the loss of clonogenicity, the induction of cell death and finally in the reduction of tumor size. This is achieved either directly or indirectly: radiation induces DNA lesions and creates highly reactive radicals that then also damage DNA. The accumulation of DNA lesions can jeopardize the genomic stability of the cell, especially when the DNA damage response (DDR) system is impaired. Individuals with germ-line mutations in DDR genes show a higher predisposition for cancer. Errors in this highly regulated process of DDR can result in accumulation of genomic mutations and malignant transformation. On the other hand, DDR also acts as a negative saboteur to resist CT and RT (5). Forms of IR-induced DNA damage that endanger chromatin integrity are single-strand breaks (SSBs) and double-strand breaks (DSBs) of the DNA, whereas SSBs (~1000/Gy) (6) are way more frequent than DSBs (~40–50/Gy) (7). However, the time to repair DSBs takes much longer. Cells have developed several DNA repair pathways, such as homologous recombination (HR), non-homologous end-joining (NHEJ), nucleotide excision repair (NER), and base excision repair (BER) as well as mismatch repair (MMR) dependent on size and modality of the DNA damage (for further interest on this topic refer to Ref. (8)). Still, if the cell is no longer able to compensate the damage, cell death is the final consequence.

## RT-Induced Cell Death

Mitotic catastrophe, apoptosis, autophagy, and senescence have been the most prominent observed forms of cell death induced by RT (9). Within the last years, it has become evident that tumor cell necrosis can be induced in a programed manner besides occurring through a more or less unregulated process (10).

### Apoptosis

**Figure 1:**
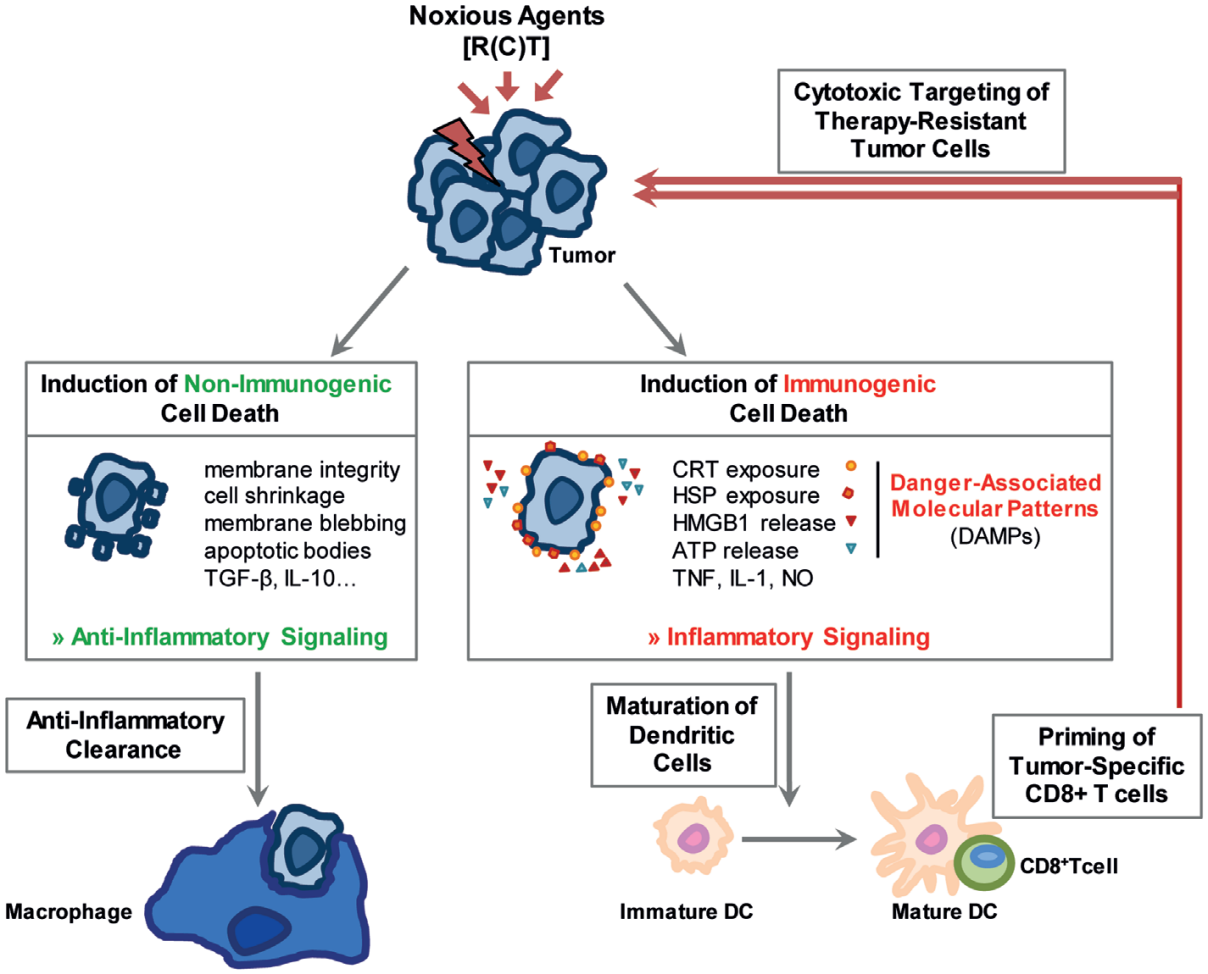
**Noxious agents may induce non-immunogenic and immunogenic cell death**. Stressed cells can either undergo a non-immunogenic cell death resulting in their anti-inflammatory clearance. The stress-resulting damage might, however, also foster immune reactions. Immunogenic forms of cell death, main characteristics of which are displayed in the figure, stimulate the immune system especially through the release of damage associated molecular patterns (DAMPs). Dendritic cells mature, are activated and initiate a cytotoxic T-cell response against the tumor cells.

Apoptosis is a programed cell suicide and the best characterized form of cell death. It is of particular importance during development and aging to maintain a homeostatic balance in tissues. A dysfunctional regulation can result in autoimmune diseases, viral infections, or cancer. In cancer therapy, apoptosis can be induced in tumor cells by the use of IR and/or cytotoxic drugs (11). In general, two main pathways exist: the intrinsic mitochondrial pathway is mainly regulated by proteins of the B-cell lymphoma-2 (Bcl-2) family, which includes pro- and anti-apoptotic proteins, whereas the extrinsic pathway is induced by cell death receptors on the cell surface. In early stages of apoptosis, the cell maintains its organelle integrity and the cell membrane remains intact. In later stages, various morphological changes of the cell are visible: cell shrinkage, chromatin condensation, DNA fragmentation, membrane blebbing, and formation of apoptotic bodies (12). Under normal conditions, apoptotic cells are engulfed by neighboring “non-professional” phagocytic cells, such as mesenchymal and epithelial cells (13). However, if the number of apoptotic cells exceeds a certain level, professional phagocytes are attracted to the site by the so-called “find me” signals that are released by dying cells (14, 15). These signals include factors such as nucleotides, proteins, and phospholipids (14, 15). The uptake of apoptotic cells by other cells is facilitated by changes within the outer membrane of the dying cell, the so-called “eat me” signals. One of the most prominent of these signals is phosphatidylserine (PS) that, under normal conditions, is located on the inner plasma membrane leaflet. During apoptosis, however, it translocates to the outer side of the lipid layer where it can be recognized by adaptor proteins and specific PS receptors on phagocytes (16). The detection and ingestion of apoptotic material by macrophages predominantly induces the release of anti-inflammatory cytokines while simultaneously inhibiting the production of pro-inflammatory cytokines (Figure 1). By contrast, the uptake of apoptotic cells by immature dendritic cells (DCs) inhibits their maturation and induces tolerance (17). Therefore, apoptosis subserves several pro-tumor functions (18). Strategies have emerged to increase the immunogenicity of apoptotic cells by blocking their clearance by macrophages with the PS-binding protein AnnexinA5 (AnxA5) (19). Pre-clinical experiments revealed a long-lasting immune memory against tumor cells and a delayed tumor growth mediated by AnxA5 when given in combination with IR (20).

Nevertheless, apoptosis often plays a subordinate role in solid tumors, as tumor cells acquire resistance to apoptosis through several mechanisms; e.g., the tumor suppressor gene p53 is mutated in more than 50% of human malignancies (9). Other resistance mechanisms are overexpression of anti-apoptotic proteins, inactivation of pro-apoptotic genes, as well as interference with the death cell receptor and perforin/granzyme pathway (21).

### Necrosis

In contrast to apoptosis, necrosis has often been defined as an uncontrolled or pathological cell death, which can be induced by extreme cellular stress such as trauma, infections, detergents, toxic agents, or heat. Morphologically, it is characterized by cellular swelling, rupture of the plasma membrane, and loss of intracellular content (22). It is considered to be a pro-inflammatory form of cell death due to its release of damage-associated molecular patterns (DAMPs) such as heat shock proteins (HSP), high mobility group box 1 (HMGB1), nucleotides, or uric acid leading to an activation of both, the innate and the adaptive immune system (22, 23) (Figure 1). In the last few years, it has become clear that there is a second form of necrosis, the so-called necroptosis, which is dependent on the receptor-interacting protein (RIP) kinases RIP1 and RIP3 (24, 25). It can be induced by factors such as tumor necrosis factor (TNF), Fas Ligand, or TNF-related apoptosis-inducing ligand (TRAIL) and utilizes the same initial signaling cascade as cell death receptor-induced apoptosis (25, 26). In addition, necroptosis can be manipulated by inhibitors such as Necrostatin 1, which blocks RIP1 kinase activity (27).

Therapeutic applications of RT and CT, either as stand-alone therapies or in combination with targeted therapies or IT, should stimulate local and systemic tumor control through the induction of immunogenic forms of cell death, which in turn can initiate persistent anti-tumor immune response. Particularly, both forms of necrosis are considered to be more immunogenic than the apoptosis and can therefore be useful tools to shift the tumor microenvironment toward an immunostimulatory rather than an immunosuppressive one (28) (Figure 1).

## Impact of the Fractionation of Radiation on Anti-Tumor Responses

It has become obvious that radiation-induced non-(DNA) targeted, systemic effects are immune mediated and therefore also in part dependent on the primary cell death induction in the irradiated area (29, 30). With the emerging development of accelerators that has made it possible to deliver precisely higher single doses into the tumor area, one should focus on the immunological consequences of the different forms of radiation treatment as current pre-clinical data are not conclusive (2). RT can be administered in conventional fractionation schemes (1.8–2.2 Gy/fraction; 1 fraction/day, 5 days/week for 3–7 weeks), hyperfractionation (0.5–2.2 Gy/fraction, two fractions/day, 2–5 fractions/week for 2–4 weeks) or hypofractionation (3–20 Gy/fraction, 1 fraction/day) (31) using various therapeutic systems, including stereotactic radiosurgery. In the latter, the external radiation procedure utilizes multiple convergent beams to deliver high single doses to a small volume while sparing adjacent normal tissue. Currently, three different main modalities, namely LINAC, Gamma Knife, and protons are used for stereotactic radiosurgery, especially for the treatment of brain tumors with limited size that cannot be removed surgically (32).

Pre-clinically, Rubner et al. showed that fractionated RT is the main stimulus for cell death induction and HSP70 release in p53 mutated and O6-methylguanine methyltransferase, a DNA repair protein, negative glioblastoma cell lines (33). Tsai et al. investigated whether single high dose vs. multiple small doses with a total dose of 10 Gy differentially alters gene expression. They found out, amongst others, that there are significant differences in the gene response depending on the fractionation of radiation: 10 Gy delivered in fractions lead to a more stable induction of genes (34). Multhoff et al. hypothesized that conventional fraction schemes over several weeks are thought to be rather negative for radiation-induced anti-tumor immune responses as tumor-infiltrating immune lymphocytes might be killed by the repeating irradiation (35). Dewan et al. investigated the effects of RT with immune modulatory anti-CTLA4-antibodies on induction of anti-tumor immune responses. In his model system 3 × 8 Gy was superior to that of 5 × 6 Gy in induction of a T-cell-dependent abscopal anti-tumor effect (36). This indicates that a higher single dose applied in hypofractionated schemes is advantageous to boost the immune system.

While it has been known that cellular effects of stereotactic radiosurgery include the induction of necrotic cell death and endothelial proliferation with luminal narrowing and thrombosis, Witham et al. used a rat glioma model to investigate whether gamma knife radiosurgery also induces apoptosis. They found tumor apoptosis to be statistically higher in treated animals at 6, 24, and 48 h after radiosurgery (37). Taken together, all these data show that more pre-clinical and clinical research are needed to define the best single dose and the respective fractionation scheme for induction of immunogenic cancer cell death and consecutive anti-tumor immunity.

## Induction of Anti-Tumor Immunity

### The role of DCs in anti-tumor immune response

The activation of the immune system is vital to promote a long-lasting anti-tumor response. An essential asset for creating a potent anti-tumor immunity is the activation of DCs and consecutively cytotoxic CD8^+^ T lymphocytes (CTL) alongside with CD4^+^ T lymphocytes. Being the most efficient antigen-presenting cells, DCs play an important role in the initiation of the adaptive immunity. However, before DCs can stimulate any other cell type, they have to be activated properly. Such activating signals are not only foreign substances or infected cells, but can also be derived endogenously from stressed cells or cells dying by necrosis (38), as it is the case in tumor therapy. Immature DCs can then acquire and process tumor material, migrate to lymph nodes, and present or cross-present peptides of tumor-associated antigens (TAA) to naïve T cells in a MHC-II- or MHC-I-dependent manner, respectively (39). Aside from stimulating T-cell responses via the expression of co-stimulatory molecules such as CD80, CD86, as well as members of the TNF family (e.g., CD40, CD137 (4-1BB)L, OX40L) that can interact with the corresponding receptors on T cells, mature DCs secrete a wide range of pro-inflammatory cytokines (40). They therefore favor T-cell activation, survival, and differentiation and thus specific anti-tumor immune responses (41, 42).

### DAMPs as mediators of DC activation

Other important factors for DC activation and maturation are secreted or exposed danger signals by dying cells, the so-called DAMPs (43). The surface exposure or release of DAMPs can be induced by IR or certain immunogenic chemotherapeutics, which are therefore capable of initiating a solid anti-tumor immune response (44). One of these signals is the early pre-apoptotic exposure of the endoplasmic reticulum (ER) protein calreticulin (CRT) on the plasma membrane surface. This can be induced by IR or substrates such as anthracyclines or oxaliplatin and triggers the uptake of tumor cells by DCs. In the presence of later DAMPs, such as HMGB1, the internalized tumor antigens get processed and cross-presented finally resulting in stimulation of tumor-specific CTLs (45, 46).

High mobility group box 1 is a chromatin-associated nuclear protein functioning as a DAMP when being expressed extracellularly. It is passively released by necrotic or damaged cells and secreted by immune cells such as macrophages, natural killer (NK) cells, neutrophils, mature DCs, and T cells and binds with high affinity to the receptor for advanced glycation end-products (RAGE) as well as the toll-like receptors (TLR)2, TLR4, and TLR9 (47). Its release from tumor cells can be induced by various stimuli, such as RT (33) and especially after combinatory treatment of RT with further immune stimulation, e.g., HT (48). Chemotherapeutic agents like temozolomide, melphalan, and paclitaxel might also foster its release (49, 50). HMGB1 interaction with a functional TLR4 on DCs is required for an efficient cross-presentation of tumor-antigens to T cells (51) and the priming of a tumor-specific T-cell response. The importance of TLR4 activation via danger signals can be seen in patients suffering from breast cancer, head and neck squamous cell carcinomas (HNSCC), or colorectal cancer carrying a loss of function single-nucleotide polymorphism (SNP) in the *Tlr-4* locus that have a predicted worsened outcome after immunogenic CT with anthracyclines or oxaliplatin (51, 52). However, HMGB1 also shows pro-tumorigenic properties. Thus, overexpression of HMGB1 and its receptor RAGE is observed in several cancers and is associated with tumor growth and metastasis (53). A possible explanation for the contradictory effects of HMGB1 might be a change of its redox state. Reducible HMGB1 binds to RAGE but not to TLR4 and promotes resistance to melphalan, paclitaxel, doxorubicin, and oxaliplatin, oxidized HMGB1, on the other hand, increases the cytotoxicity of these agents (54). One might speculate that RT-induced mitochondrial ROS production contributes to oxidation of HMGB1 and thereby to immunogenicity (55).

Another example for a DAMP that can be either passively released or actively secreted by dying or stressed cells is adenosine-5-triphosphate (ATP). It acts on purinergic P2RX7 receptors on DCs that in turn activate the NLRP3/ASC/caspase-1 inflammasome, finally resulting in the secretion of interleukin (IL-) IL-18 and IL-1β (56). IL-1β is required for efficient priming of CD4^+^ T cells and interferone-γ (IFN-γ) producing tumor antigen-specific CD8^+^ CTLs (57) and therefore for the generation of an anti-tumor immune response. Furthermore, ATP release from tumor cells also contributes to tumor growth and modulates immunosuppressive properties of myeloid-derived suppressor cells (MDSC) via a P2 × 7 receptor dependent mechanism (58).

HSP70 released from stressed cancer cells can also serve as a danger signal. HSPs are among the most abundant proteins in cells. Intracellular HSPs function as chaperons ensuring the correct folding or degradation of misfolded proteins. Under stress-induced conditions such as oxidative stress, HT, irradiation, or chemotherapeutics, intracellularly located HSPs are overexpressed and can be translocated to the plasma membrane or be released into the extracellular compartment, thereby acting as danger signals. In this way, HSP70 and HSP90 in particular play a dual role in cancer. Intracellularly, they protect tumor cells from programed cell death by interfering with apoptotic processes (59). However, if they are bound to the plasma membrane or released they contribute to the activation of the innate and adaptive immune system (60, 61). HSP70 promotes DC maturation as well as NK cell migration, activation, and cytolytic activity. Also HSP70 is thought to be associated with tumor antigens triggering their cross-presentation via MHC-I on DCs and stimulating a CD8^+^ T-cell response (62). Relevance of exposed HSP70 as a tumor-specific recognition structure is given by the group of Multhoff et al. who found that HSP70 is expressed on the plasma membrane of 40 (colon), 37 (gastric), 43 (lower rectal), and 42% (squamous cell) tumor specimens, but never on healthy cells. However, during the investigation, it became clear that the tumor entity is of major importance for clinical outcome. They therefore suggest the usage of HSP70 as a potential prognostic marker for overall survival (OS) (63).

To sum up, danger signals such as CRT, HMGB1, ATP, and HSPs are inducible by several chemotherapeutic drugs or irradiation. They play important roles in the priming of anti-tumor immune responses, but, depending on their location, concentration, and redox state, can also promote tumor development and progression.

## Therapy-Dependent Modulation of the Tumor Microenvironment

Tumors have developed several molecular and cellular mechanisms to evade immune surveillance. These strategies include the secretion of immunosuppressive factors such as TGF-β, IL-10, or indoleamine 2,3-dioxygenase (IDO) (64–68), the alteration of antigen-presentation (69, 70), disruption of T-cell activation (71), apoptosis promotion of activated T cells (72), as well as the recruitment of regulatory cells or in general the inhibition of immune cells (73–75).

However, given that the immune system provides a possible strategy to create an efficient and long-lasting anti-tumor response, it is necessary to find treatment strategies that overcome the protective immunosuppressive microenvironment created by the tumor. Lately, it has become clear that standard treatments, namely RT and CT, can already render tumors and their microenvironment more immunogenic (76). As outlined above, RT and CT are able to induce both apoptotic and necrotic tumor cell death resulting in surface exposure and release of danger signals or TAAs. Aside from inducing tumor cell death, various chemotherapeutics, even or especially at low concentrations, stimulate, e.g., the expression of components of the antigen-processing machinery together with co-stimulatory molecules (e.g., CD40, CD80, CD86, MHC-II) on DCs thus promoting the stimulation of tumor-specific T cells, resulting in an anti-tumor immune response.

### Immunogenicity of radiotherapy

While low doses of IR have anti-inflammatory effects (77), higher doses (>1 Gy) applied in tumor therapy are capable of stimulating the immune system in several ways: RT can enhance the expression of MHC-I on the surface of tumor cells alongside with cell death receptors Fas/CD95 and NKG2D ligand, thus boosting the recognition and killing of irradiated tumor cells through T cells and NK cells (78–80). IR also has the ability to induce the production and release of CXCL16 in tumor cells. CXCL16 is a chemokine binding to its receptor CXCR6 on activated T cells therefore enhancing their recruitment to the tumor site (81). In addition, it also increases IFN-γ production that alters expression of adhesion molecules on vasculature, chemokines, and MHC-I expression, thus creating a microenvironment beneficial for T-cell infiltration and recognition of tumor cells by CTLs (82). Both, fractionated, hypofractionated, and ablative regimes bear the potential to stimulate immune responses (83, 84). However, which fractionation scheme and single dose of RT is the most immunogenic is under current intensive investigation and discussion (15, 42).

Taking all these factors into account, it becomes clear that CT and RT aside from their initial purpose to stop the proliferation of tumor cells and kill them are useful tools to shift an immunosuppressive tumor microenvironment to a more beneficial immune stimulatory one. A detailed understanding of the molecular mechanisms underlying these effects is therefore essential toward an optimized treatment.

## Systemic Effects of Radiotherapy

As mentioned before, radiation, together with surgery and chemotherapeutics, is one of the most important tools in cancer treatment with the primary goal to achieve local control of tumor growth. Furthermore, it also enhances the tumors immunogenicity through the induction of distinct tumor cell death forms and the release of pro-inflammatory cytokines, chemokines, as well as danger signals. It therefore bears the potential to induce adaptive and innate immune responses, resulting in systemic anti-tumorigenic effects even outside of the field of irradiation (85). The phenomenon of regression of distant tumors or metastases outside the irradiation field is called *abscopal effect* of RT and its connection with immune events was first described by Nobler in 1969 (86). Since abscopal sounds a bit mystic, one should rather term it *systemic immune-mediated* effects of RT nowadays. Such reactions have been observed in many pre-clinical studies as well as in clinical settings for several tumor entities, including melanoma, hepatocellular, renal-cell, and mammary carcinomas, chronic lymphocytic leukemia (CLL), or malignant lymphomas [for further reading, see Ref. (42, 87)].

On a cellular level, it was demonstrated that the adaptive immune systems contributes to these systemic reactions and that NK cells are also involved (88, 89). In addition, the release of danger signals or cytokines such as TNF-α and IFN-γ by radiation-damaged tumor cells promote DC maturation and cross-presentation resulting in the regression of more distant tumor masses through activation of tumor-specific T cells (36, 88, 90).

However, in most tumor entities RT alone is not sufficient to induce such systemic immune reactions (89). Therefore, combination with IT might be the solution. A combined treatment of RT with the DC growth factor Flt-3 induced immune-mediated anti-tumor responses outside the irradiation field (89). Shiraishi et al. observed such effects after combined treatment of colon26 adenocarcinoma-bearing Balb/c mice with fractionated RT and the macrophage inflammatory protein-1 alpha variant ECI301 (88). A better local control and regression of the not irradiated tumor was observed by Jurgenliemk-Schulz and colleagues after additional rIL-2 treatment to RT in SL2 lymphoma or M8013 mammary carcinoma inoculated mice (91). Another approach is the modification of tumor cells or DCs with genetically engineered viruses expressing various cytokines, including IL-2 (92, 93), IL-12, IL-18 (94, 95), GM-CSF (96), or IFN-β (97) to enhance anti-tumor immunity and protect against tumor re-challenge. Just recently, Golden et al. reported about immune-mediated systemic tumor responses when combing RT with GM-CSF for the treatment of patients with metastatic solid tumors (98).

A further encouraging strategy to improve the effectiveness of standard therapies is the usage of monoclonal antibodies (mAb) targeting immune cells or tumors. In this matter, therapeutic Ab (summarized in Table 1) that can be used either alone or in combination with RT, CT, or IT are involved in depletion of Tregs (anti-CD25) (99) or target (i) co-stimulatory molecules such as CD40 (100, 101), OX40 (CD134) (102), and 4-1BB (CD137) (103, 104) on immune cells; (ii) checkpoint inhibitors PD-1, PD-L1 (103, 105) and CTLA-4 (99, 104); and (iii) cell growth factors or their receptors, e.g., epidermal growth factor receptor (EGFR), vascular endothelial growth factor (VEGF), and VEGFR (106, 107), all of which will be discussed in the following paragraph.

**Table 1:** **Selected monoclonal antibodies and tyrosine kinase inhibitors against co-stimulatory and checkpoint molecules and growth factors that are in clinical phase I–III trials either alone or in combination with RT, CT or immunotherapy**.

Target	Drug	Developer	Target disease (not all listed)
**Co-stimulatory molecules**			
CD40	CP-870,893 Dacetuzumab Lucatumumab	Pfizer Seattle Genetics, Inc. Novartis	Melanoma; pancreatic carcinoma; *renal-*cell carcinoma; breast cancer Diffuse large B-cell lymphoma (DLBCL); chronic lymphocytic leukemia (CLL); non-hodgkin’s lymphoma (NHL); multiple myeloma (MM) CLL; NHL; MM
CD134 (OX40)	MEDI6469	AstraZeneca	Advanced solid tumors; aggressive B-cell lymphomas; HNC; metastatic prostate cancer
CD137	BM-663513	Bristol-Myers Squibb (BMS)	Melanoma; advanced solid malignancies; B-cell malignancies
**Checkpoint inhibitors**			
CTLA-4	Tremelimumab Ipilimumab	Pfizer BMS	Metastatic melanoma; HNSCC; NSCLC; advanced solid malignancies Yervoy™ approved for unresectable or metastatic melanoma^a^; lymphoma; NSCLC; HNC; prostate, pancreatic, liver, lung, kidney and renal-cell cancer; melanoma
PD-1	Nivolumab Pembrolizumab Pidilizumab	BMS Merck CureTech Ltd	Obvido^®^ approved for unresectable or metastatic melanoma and NSCLC^a^; MM; NHL Renal-cell carcinoma (RCC); advanced solid tumors; melanoma; NSCLC Keytruda^®^ approved for advanced or unresectable melanoma^a^; NSCLC; HNSCC; lymphoma; breast cancer; malignant glioma; melanoma MM; gliomas; lymphomas
PD-L1	BMS-936559 MEDI4736	BMS AstraZeneca	Recurrent solid tumors Advanced solid tumors; NSCLC; HNSCC; GBM
**Growth factor inhibitors**			
EGFR	Cetuximab Panitumumab Gefitinib Erlotinib	BMS Amgen AstraZeneca Genentech/Roche	Erbitux^®^ approved for *K-ras* wild-type, EGFR-expressing metastatic colorectal cancer and recurrent/metastatic HNSCC^a^; NSCLC; HNSCC; colorectal cancer Vectibix™ approved for colorectal cancer^a^; HNSCC; colorectal cancer Iressa^®^ approved for NSCLC^a^; HNC; skin, breast, colorectal cancer; GBM; NSCLC Tarceva^®^ approved for NSCLC and pancreatic cancer^a^; HNC; prostate, breast, esophageal, colorectal cancer; NSCLC; pancreatic cancer
HER2/neu receptor	Trastuzumab	Genentech/Roche	Herceptin^®^ approved for HER2-overexpressing breast cancer and HER2-overexpressing metastatic gastric or gastroesophageal (GE) junction adenocarcinoma^a^; breast cancer; NSCLC
VEGFRs, PDGFRs, FLT-3, c-Kit, RET; CSF-1R	Sunitinib	Pfizer	Sutent^®^ approved for pancreatic neuroendocrine tumors (pNET); kidney cancer and gastrointestinal stromal tumor (GIST)^a^; pNET; kidney cancer; GIST; RCC, pancreatic and bladder cancer
VEGFRs, PDGFRs, RAF, FLT-3, c-Kit, RET	Sorafenib	Bayer	Nexavar^®^ approved for recurrent or metastatic, progressive differentiated thyroid carcinoma (DTC), unresectable hepatocellular carcinoma (HCC) and advanced RCC^a^; HCC; RCC, bladder cancer; brain neoplasms; advanced solid tumors
VEGFRs	Axitinib	Pfizer	Inlyta^®^ approved for advanced RCC^a^; advanced gastric cancer; hepatocellular and colorectal carcinoma; prostate cancer; GBM; RCC; NSCLC
VEGFRs, PDGFRs, c-Kit	Pazopanib	GlaxoSmithKline	Votrient^®^ approved for advanced soft tissue sarcoma and RCC^a^; ovarian cancer; fallopian tube cancer; peritoneal carcinoma; NSCLC; RCC
VEGF-A	Bevacizumab	Genentech/Roche	Avastin^®^ approved for recurrent epithelial ovarian, fallopian tube, or primary peritoneal cancer, recurrent/metastatic cervical cancer, metastatic HER2 negative breast cancer, RCC, GBM, NSCLC^a^; advanced cancers

*^a^FDA-approved drugs*.

## Co-Stimulatory Molecules as Target to Improve RT and CT-Induced Systemic Immune Responses

### CD40

CD40, a member of the TNF receptor (TNF-R) family, is expressed on APCs such as DCs, B cells, and macrophages. Interaction with its ligand (CD40L) on activated T cells promotes their activation and subsequently the induction of adaptive immune responses. Furthermore, the interaction between CD40 and its natural ligand (CD40L, CD154) was shown to modulate the growth of malignant B cells, thus CD40-related therapies have been considered for a range of cancer entities, including B-cell malignancies, leukemia, and multiple myelomas (MMs) (108), making it an attractive target structure. CD40 agonists mediate tumor cell death and in combination with DC activation anti-tumor immune responses. Pre-clinical models showed that anti-CD40 therapy in combination with RT results in a CD8 T-cell-dependent immunity to B-cell lymphoma (101). Currently, there are several anti-CD40 antibodies such as CP-870,893, dacetuzumab, and lucatumumab either as stand-alone treatments or in various combinations under investigation, such as a phase 1A/II study (NTC00670592) of patients with advanced non-Hodgkin lymphoma (NHL) or Hodgkin lymphoma (HL), which demonstrated a modest lucatumumab activity (109). However, there is still a lack of clinical data assessing the efficacy of targeting CD40 especially in combinatory therapy regimens with RT, CT, and other ITs, which is why further investigations are necessary.

### OX40

OX40 (CD134), a co-stimulatory molecule expressed on activated T cells, is also part of the TNF-R superfamily. A phase I trial (NCT01644968), focusing on anti-OX40 monotherapy with a murine agonistic anti-human Ox40 mAb (9B12) in patients with metastatic solid malignancies showed an increased proliferation of CD4^+^/FoxP3- and CD8^+^ T cells as well as CD3-/NK cells. While anti-OX40 treatment was well tolerated with mild to mediate side effects, 12 out of 30 patients showed regression of at least one metastatic lesion (110). In order to increase this effect, a variety of combinatory therapy strategies of anti-OX40 treatment with CT, RT, or other IT are currently under investigation. For instance, a murine model of stereotactic body radiation therapy (SBRT) of lung cancer showed significant enhancement of tumor-free survival through intensified tumor antigen-specific CD8^+^ T-cell responses under RT combined with adjuvant anti-OX40 therapy (111). Furthermore, a phase I/II trial with SBRT plus anti-OX40 in patients suffering from metastatic breast cancer (NCT01862900) and a phase Ib trial with cyclophosphamide, RT, and anti-OX40 in patients with progressive metastatic prostate cancer (NCT01303705) are currently ongoing with results not yet published.

### CD137 (4-1BB)

CD137, expressed on activated CD4^+^ and CD8^+^ T cells, as well as on several APCs, including DCs, activated B cells, and macrophages, co-stimulates T-cell activation and clonal expansion after T-cell receptor (TCR) engagement through interactions with CD137-ligand. Importantly, the therapeutic use of 4-1BB agonists *in vivo* leads to a biased CD8^+^ T-cell activation with a concomitant decline of B cells, NK, and CD4^+^ T cells in an IFN-, TNF-, TGF-β, and IDO-dependent fashion (112). Furthermore, stimulation of CD137 on tumor endothelial cells via an agonistic antibody upregulates ICAM1, VCAM1, and E-selectin and thereby enhances T-cell recruitment into tumor tissue (113). In murine lung (M109) and breast carcinoma (EMT6) models, the efficiency of BMS-469492, another agonistic CD137 mAb, in combination with RT was evaluated. In the case of lung carcinoma treatment only a combination of the antibody with RT administered as a high radiation dose of 15 Gy resulted in an enhanced tumor response. In the breast cancer model, the CD137 agonist alone already led to significant tumor growth inhibition that could even be potentiated by using high single doses or fractionated radiation. The authors concluded that the different responses in the two models could result from differences in intrinsic immunogenicity of the different tumor entities and that anti-CD137 antibodies may not only be used as a stand-alone therapy but in combination with conventional anti-cancer treatments, e.g., RT (114). Furthermore, the combination of RT and anti-CD137 in an intracranial glioma model resulted in complete tumor elimination and prolonged survival in 67% of the mice. The combination therapy highly increased the numbers of tumor-infiltrating CD4^+^ and CD8^+^ lymphocytes as well as IFN-γ production (115). Thus, based on promising pre-clinical data of combining anti-CD137 and RT/CT, two currently ongoing clinical phase I studies have been initiated. While NCT00461110 investigates agonistic anti-CD137 (BMS-663513) treatment in combination with chemo-radiation (RT, paclitaxel, carboplatin) in non-small cell lung carcinoma (NSCLC) patients, NTC00351325 focuses on a combination therapy of BMS-663513 with CT (paclitaxel, carboplatin) in patients suffering from recurrent ovarian carcinoma.

## Checkpoint Inhibitors as Targets to Improve RT-Induced Systemic Immune Responses

In order to ensure peripheral tolerance and to avoid overshooting immune reactions, endogenous mechanisms to dampen T cells have been evolved. Cytotoxic T lymphocyte-antigen-4 (CTLA-4) and PD-1 are major negative co-stimulatory molecules expressed on activated T cells (116–118). While CTLA-4 regulates early stages of T-cell activation, PD-1 limits the activity of T cells in peripheral tissues during inflammatory response and is therefore a major immune resistance mechanism in the tumor microenvironment.

### CTLA-4

T-cell activation and survival are dependent on positive signaling from the TCR as well as co-stimulatory molecules such as CD28. CTLA-4 is an inhibitory molecule that is upregulated on the surface of effector T cells and competes with CD28 for the binding to CD80/86 (B7.1 and B7.2). Under physiological conditions, this limits the T-cell response and helps to maintain T-cell homeostasis (119). However, with regard to cancer treatment, the down-regulation of a tumor-specific T-cell response is an unwanted scenario, thus favoring an antagonistic CTLA-4 therapy. Indeed, various pre-clinical and clinical studies have already proven the efficiency of anti-CTLA-4 therapy, especially for melanoma in multimodal settings. This tumor entity has a high prevalence for somatic mutations (120) and is therefore suitable for being specifically targeted by activated immune cells.

Pre-clinical melanoma models showed that tumors do not always respond to an anti-CTLA-4 mAb alone, while additive treatments with GM-CSF (121) or activation of the T-cell co-stimulatory receptor 4-1BB (122) are able to promote an anti-tumor response. Furthermore, a combination of RT and CTLA-4 mAb treatment prolonged the OS in an orthotopic GL261 glioma model, whereas CTLA-4 mAb alone was not able to extend the survival in comparison to untreated controls. A triple combination of RT, anti-CTLA-4, and anti-CD137 further improved survival in this pre-clinical model through a CD4^+^ T-cell-dependent manner and created a glioma-specific protective memory response (104). Dewan and colleagues reported an abscopal effect in breast (TSA) and colon cancer (MCA38) models: An increased frequency of CD4^+^ and CD8^+^ tumor-infiltrating lymphocytes along with tumor-specific IFN-γ production was observed after a combined administration of anti-CTLA-4 mAb (9H10) and fractionated (3 × 8 Gy or 5 × 6 Gy fractions in consecutive days), but not single-dose RT with 20 Gy. Furthermore, three doses of 8 Gy in combination with anti-CTLA-4 was able to induce a more potent systemic effect and higher frequency of tumor-specific T cells than five doses of 6 Gy plus anti-CTLA-4 (36), suggesting that fractionation influences the induction of anti-tumor immune responses with further immune stimulation (76).

Two fully humanized anti-CTLA-4 antibodies, tremelimumab and ipilimumab, advanced for testing in clinical trials. Most studies so far are focused on melanoma, where treatment-related adverse effects were found to be manageable (123–125). Various phase I and II studies evaluated anti-CTLA-4 therapy in a stand-alone therapy setting or in various combinations such as tumor antigen-loaded DCs (126, 127), the TLR9 agonist PF-3512676 (128), IFN-α-2b (129) or in combination with various chemotherapeutics (for further reading, refer to NCT00257205 (130), NCT02262741, NCT02319044, NCT02369874, NCT02352948, NCT02040064).

In summary, current study results show the importance of investigating the optimal dose, schedule, and combination of anti-CTLA-4 antibodies with other therapy options to ensure high patient safety and efficacy in selected cancer entities.

As approximately 50% of cancer patients receive RT with the primary goal of local tumor control (4), combinatory therapies of RT with immune checkpoint inhibitors targeting T cells might be a good synergistic option to induce additional systemic anti-tumor immune responses, as it has already been shown in many mouse models (Table 2) (36, 131–134). A tremelimumab/SBRT pilot study for patients suffering from unresectable pancreatic cancer (NCT02311361) is currently recruiting patients.

**Table 2:** **Systemic effects observed in pre-clinical and clinical studies after multimodal treatment of RT, CT, and immunotherapy**.

Checkpoint	Tumor type	Treatment	Systemic effects + key mediator	Reference
**PRECLINICAL MOUSE-MODELS**				
CTLA-4	Metastatic mammary carcinoma (4T1)	RT (2 × 12 Gy) of primary tumor + anti-CTLA-4 mAb i.p. (3×)	Inhibition of lung metastases, ↑**CD8^+^ CTLs**	(131)
	Metastatic mammary carcinoma (4T1)	RT (2 × 12 Gy) of primary tumor + anti-CTLA-4 (9H10) mAb i.p. (3×)	Inhibition of lung metastases, increased survival, ↑**CD8^+^ CTLs**	(132)
	Mammary carcinoma (TSA), colon carcinoma (MCA38)	RT of primary tumor (20 Gy, 3 × 8 Gy, 5 × 6 Gy) + anti-CTLA-4 (9H10) mAb i.p. (3×)	Growth-inhibition of irradiated and non-irradiated tumor, ↑**CD8^+^ CTLs and CD4^+^ Th-cells, IFNγ**	(36)
PD-1	Melanoma (B16), renal cortical adenocarcinoma (RENCA)	SABR (15 Gy) + anti-PD-1 mAb (5×)	Near-complete regression of primary tumor, 66% size reduction of non-irradiated tumor,↑**CD8^+^ CTLs**	(133)
	Glioma (GL261)	RT (10 Gy) + anti-PD-1 mAb i.p.	Tumor regression and long-term survival, **↓ Tregs**, ↑ CD8^+^ CTLs, IFNγ	(105)
	Melanoma (B16), breast carcinoma (4T1-HA)	RT (12 Gy) + anti-PD-1 mAb i.p. (3×)	Tumor regression and tumor control, **↓ Tregs, ↑ CD8^+^ CTLs**	(135)
PD-L1	Mammary carcinoma (TUBO)	SABR (12 Gy) + anti-PD-L1 mAb (4×)	Size reduction of primary and abscopal tumors, **↓ MDSC, ↑ CD8^+^ T-cells**	(134)
CD137 (4-1BB)	Lung carcinoma (M109)	RT (5, 10 or 15Gy) + anti-CD137 (BMS-469492) mAb i.v. (3×)	Tumor growth retardation at a dose of 15 Gy	(114)
	Breast carcinoma (EMT6)	RT (5, 10, 15Gy, 11 × 4 Gy) + anti-CD137 (BMS-469492) mAb i.v. (3×)	Enhanced tumor growth retardation at all radiation doses	(114)
	Glioma (GL261)	RT (2 × 4 Gy) + anti-CD137 mAb i.p. (3×)	Tumor eradication, prolonged survival (6/9), rejection of challenging tumors (5/6), ↑**CD8^+^ CTLs and CD4^+^ Th-cells, IFNγ**	(115)
CTLA-4 + CD137	Glioma (GL261)	RT (10 Gy) + anti-CD137 and anti-CTLA-4 mAb i.p. (3×)	Prolonged survival, ↑**CD8^+^ CTLs and CD4^+^ Th-cells**	(104)
**CLINICAL STUDIES**				
**Checkpoint inhibitors**				
CTLA-4	Metastatic melanoma (*n* = 1)	RT (28.5 Gy in 3 fractions) + ipilimumab	Regression of irradiated and non-irradiated tumor lesions, stable lesions and minimal disease 10 months after RT	(136)
	Metastatic melanoma (*n* = 1)	RT (54 Gy in 3 fractions) + 4 cycles of ipilimumab	Regression of irradiated and non-irradiated tumor lesions, CR, no evidence of disease 12 months after RT	(137)
	Melanoma with brain metastasis (*n* = 21)	Four cycles of ipilimumab + loco-regional RT	13/21 LR, 11/21 with LR abscopal effect and 2/21 stable disease	(138)
	mCRPC (*n* = 799) [NCT00861614]	RT (1 × 8 Gy) per lesion + 1–4 doses of ipilimumab (*n* = 399) or placebo (*n* = 400)	Improved median OS	(139)
	Metastatic NSCLC (*n* = 1)	RT (5 × 6 Gy) + four cycles of ipilimumab	Regression of irradiated and non-irradiated tumor lesions	(140)
PD-1	Melanoma, NSCLC, mCRPC, colorectal cancer, and renal cancer (*n* = 236)	nivolumab	Cumulative response rates in 14/76 among NSCLC patients, in 26/94 of melanoma patients and in 9/33 renal-cell cancer patients	(141)
	Advanced melanoma	Pembrolizumab (lambrolizumab; MK-3475)	52% response rate drug-related adverse effects were reported by 79% of patients, with 13% reporting grades 3 and 4 secondary effects	(142)
	Patients with DLBCL undergoing AHSCT [NCT00532259]	AHSCT + 3 doses pidilizumab	At 16 months, PFS was 0.72, among the 35 patients with measurable disease after AHSCT, overall response rate was 51%, ↑ **circulating lymphocyte subsets including PD-L1-bearing lymphocytes**	(143)
PD-L1	Dose-escalation study in patients with NSCLC, melanoma, colorectal, renal-cell, ovarian, pancreatic, gastric, and breast cancer (*n* = 207) [NCT00729664]	Administration of BMS-936559 in 6-week cycles; up to 16 cycles	Objective response rate in 9/52 in melanoma, in 2/17 in renal-cell cancer, in 5/49 in NSCLC, and in 1/17 in ovarian cancer	(144)
**Growth factor inhibitors**				
VEGF-A	Advanced nasopharyngeal carcinoma (*n* = 44) [NCT00408694]	IMRT (50–70 Gy) + CT + concurrent and adjuvant BEV	Localregional PFS (83.7%) and distant metastasis-free interval (90.8%), PFS (74.7%), OS (90.9%) within 2 years median followup	(145)
	Advanced colorectal carcinoma (*n* = 19)	RT (15x–3.4 Gy) + concurrent and adjuvant BEV + CT	CR (68.5%) and PR (21.1%) within 2 years median follow	(146)
	Newly diagnosed GBM [NCT00943826]	RT (60 Gy) + concurrent and adjuvant TMZ + BEV (*n* = 458) or placebo (*n* = 463)	Improved PFS	(147)
	Newly diagnosed GBM (*n* = 621) [NCT00884741]	RT (60Gy) + concurrent and adjuvant TMZ + BEV or placebo	Improved PFS	(148)
EGFR	LA-HNC [NCT00004227]	RT with concurrent cetuximab (*n* = 211) or RT alone (*n* = 213)	Improved median OS	(149)
	Unresectable LA-SCCHN (*n* = 60) [NCT00096174]	RCT with concurrent and adjuvant cetuximab	Improved median OS in HPV(+) tumors	(150)
	Esophageal cancer *[ISRCTN47718479]*	RCT with cetuximab (*n* = 129) or RCT alone (*n* = 129)	↓ Survival in cetuximab group	(151)
	Unresectable NSCLC [SWOG 0023]	RCT with adjuvant gefintinib (*n* = 118) or placebo (*n* = 125)	↓ Survival in gefinitib group	(152)
	LA-HNC (*n* = 66)	CRT + concurrent and adjuvant gefintinib	CR (90%), PFS (72%), OS (74%) within 3.5 years median followup	(153)
	Metastatic NSCLC (*n* = 24)	SBRT + CT with neoadjuvant, concurrent and adjuvant erlotinib	Improved PFS and OS	(154)
	Advanced cervical cancer (*n* = 36)	RCT with neoadjuvant, concurrent erlotinib	Improved PFS and OS	(155)
	Lung adenocarcinoma with brain metastases	WBRT with concurrent and adjuvant erlotinib (*n* = 23) or WBRT alone (*n* = 21)	Median local PFS 6.8 vs. 10.6 month (mOS: 6.8 vs. 10.6 month, response rate 54.84 vs. 95.65%) in RT vs. RT + E	(156)
	Newly diagnosed GBM (*n* = 65)	RCT with concurrent and adjuvant erlotinib	Median PFS 8.2 vs. 4.9 month (mS: 19.3 vs. 14.1 month) RCT + E vs. historical controls (only RCT)	(157)
EGFR + VEGF-A	LA-HNC (*n* = 27) [NCT00140556]	Neoadjuvant BEV and/or erlotinib concurrent CRT + BEV and erlotinib	CR (96%), local control (85%) and distant metastasis-free survival rate (93%), PFS (82%), OS (86%) within 3 years median followup	(158)
VEGFR, PDGFR, KIT, RAF	Advanced hepatocellular carcinoma (*n* = 40)	RT with concurrent and adjuvant Sorafenib (S)	No improved efficacy of RT + S compared to RT alone	(159)
	Newly diagnosed GBM (*n* = 47)	RCT with adjuvant sorafenib (S)	No improved efficacy of RCT + S compared to RCT alone	(160)
RTK inhibitor	Patients with oligometastases (*n* = 25) [NCT00463060]	Sunitinib + IGRT (10 × 5 Gy)	Local (75%) and distant (52%) tumor control, PFS (56%), OS (71%) within 18-month median followup	(161)
	Patients with oligometastases (*n* = 46)	Sunitinib + SBRT (10 × 5 Gy)	Local (75%) and distant (40%) tumor control, PFS (34%), OS (29%) within 4-year median followup	(162)
**Co-stimulatory molecules**				
CD40	Advanced NHL (*n* = 74)or HL (*n* = 37) [NTC00670592]	Escalating doses of lucatumumab (once weekly for 4 weeks of an 8-week cycle)	Modest activity in relapsed/refractory patients with advanced lymphoma	(109)

*↑, increase; ↓, decrease; NSCLC, non-small cell lung carcinoma; mCRPC, metastatic castration-resistant prostate cancer; GBM, glioblastoma multiforme; LA-HNC, locally advanced head and neck cancer; LA-SCCHN, locally advanced squamous cell head and neck cancer; DLBCL, diffuse large B-cell lymphoma; NHL, non-Hodgkin lymphoma; HL, Hodgkin lymphoma; SBRT, stereotactic body radiation therapy; SABR, stereotactic ablative RT; IMRT, intensity modulated radiation therapy; IGRT, image-guided radiotherapy; WBRT, whole brain radiotherapy; AHSCT, autologous hematopoietic stem-cell transplantation; OS, overall survival; PFS, progression-free survival; CR, complete response; PR, partial response; LR, local response; BEV, bevacizumab; R-ICE, rituximab, ifosfamide, carboplatin and etopside; MDSCs, myeloid-derived suppressor cells*.

Another anti-CTLA-4 mAb is ipilimumab, as with tremelimumab, patients with advanced metastatic cancer can benefit from it. Adverse side effects such as strong autoimmune reactions have been observed in a dose-dependent manner in various phase I/II trials (163–165). An increase of the ipilimumab-induced response rate might be achieved through a combination with immunogenic RT. Postow et al. described the first case of a systemic immune-mediated effect in a patient suffering from metastatic melanoma that has been treated with ipilimumab and a concomitant palliative RT (28.5 Gy in three fractions) that correlated with beneficial immune changes in the peripheral blood when RT was added (136). Five months after RT and an additional administered ipilimumab dose, RT-treated and non-RT-treated lesions had regressed and remained stable with minimal disease progression after 10 months, as confirmed by computed tomography scans. A second case of complete systemic response after a combined treatment of ipilimumab followed by high-dose stereotactic RT of 54 Gy in three fractions to two out of seven metastatic liver lesions was reported in a patient with advanced melanoma (137). Several studies have also provided evidence of ipilimumab effectiveness in cases of melanoma with brain metastases (165, 166). Hence, in a retrospective study of 21 patients suffering from advanced melanoma and brain metastases, Grimaldi and colleagues (138) reported abscopal responses in 52% of patients receiving an initial ipilimumab therapy followed by localized RT. Furthermore, this systemic response was correlated with prolonged OS.

These promising results of combined ipilimumab and RT treatment spiked the interest and led to the initiation of studies for other cancer entities than melanoma. Likewise, a phase I/II study in patients with metastatic castration-resistant prostate cancer (mCRPC) suggests the induction of clinical anti-tumor activity with disease control and manageable adverse effects after 10 mg/kg of ipilimumab and RT with 8 Gy per lesion (167). A phase III trial (NCT00861614) evaluating ipilimumab administration (10 mg/kg) vs. placebo after RT in patients suffering from mCRPC with disease progress after docetaxel reported an improvement of median OS within the ipilimumab group (11.2 vs. 10 months in the placebo group). Conversely, most of the common adverse effects (26 vs. 3%) and four deaths occurred in patients receiving ipilimumab treatment vs. placebo (139). Just recently, a systemic response was reported in a patient suffering from metastatic NSCLC 2.5 months after the start of a combined ipilimumab and fractioned RT (140). This suggests that a combination of local RT alongside IT could be a useful approach to further improve clinical outcome of patients with metastatic disease (2). Therefore, various phase I/II studies of combined RT and ipilimumab administration for metastatic NSCLC (NCT02221739), advanced cervical cancer (NCT01711515), metastatic cancers of liver and lungs (NCT02239900), and patients with melanoma and brain metastases (NCT02115139) have been initiated and are currently recruiting patients.

### PD-1/PD-L1 (B7-H1)

PD-1, another negative regulator of TCR signaling, and its ligand PD-L1 play an important role in modulating T-cell activity not only in physiological conditions but also in the tumor microenvironment of various cancer entities. Thus, blockage of PD-1 and PD-L1 interaction through mAb is a promising strategy to overcome tumor-escape from a tumor-specific immune response (168, 169).

Pre-clinical studies have demonstrated an enhancement of anti-tumor immunity through a combination of RT together with antibody-mediated PD-1 blockade (133–135). For instance, the effectiveness of treatment of mouse melanoma and renal-cell tumors with stereotactic ablative radiotherapy (SABR) was dependent on PD-1 expression. Only 20% of PD-1 KO mice and none of the wild-type mice survived beyond 40 days. The combination of SABR with PD-1 blockade resulted not only in an almost complete regression of the irradiated primary tumor, but also in a 66% size reduction of the non-irradiated secondary tumors. Park et al. therefore suggest a SABR-induced systemic tumor-specific immune response also targeting the secondary non-irradiated tumors that can further be increased by PD-1 blockade (133). Of note is that optimal timing of RT in combination with a checkpoint blockade is mandatory: since IR temporarily increases the expression of PD-1L on tumor cells (134), a concurrent application is suggested. Addition of anti-PD-L1 mAb after RT does not result in prolonged survival of tumor-bearing mice (170, 171).

Single-agent trials have already been initiated using the anti-PD-1 mAbs nivolumab, pembrolizumab, and pidilizumab, as recently summarized by Philips and Atkins (172). Those studies include planned or ongoing phase I–II trials of anti-PD-1 mAb monotherapy for various cancer entities, such as lymphoma (NCT02038946, NCT02038933, NCT01953692), NSCLC (NCT02066636, NCT01840579), hepatocellular carcinoma (HCC) (NCT01658878), HNSCC (NCT02105636), melanoma (NCT02374242, NCT01844505, NCT02306850), and glioma (NCT02337686, NCT02359565, NCT01952769), respectively, either alone or in comparison to CT or IT. A phase I trial investigating safety and reactivity of nivolumab in 236 patients with melanoma, NSCLC, mCRPC, colorectal cancer, and renal cancer concluded cumulative response rates in 14 of 76 among NSCLC patients, in 26 of 94 of patients suffering from melanoma and in 9 of 33 renal-cell cancer patients. Anti-PD-1 treatment-related toxic effects (grades 3 and 4) occurred in 14% of the patients (141). A phase I trial with BMS-936559, a PD-L1-specific Ab in NSCLC, melanoma, colorectal, renal-cell, ovarian, pancreatic, gastric, and breast cancer (NCT00729664) patients resulted in an objective response rate in 9 of 52 in melanoma, in 2 of 17 in renal-cell cancer, in 5 of 49 in NSCLC, and in 1 of 17 in ovarian cancer, while drug-related adverse effects of grades 3 and 4 occurred in 9% of treated patients (144). A clinical investigation of lambrolizumab (MK-3475) in patients with advanced melanoma showed a 52% response rate. However, during the treatment drug-related adverse effects were reported by 79% of patients, with 13% reporting grades 3 and 4 secondary effects (142). These investigations lead to a FDA approval of pembrolizumab (formerly MK-3475 and lambrolizumab) in patients suffering from advanced or non-resectable melanoma that are no longer responsive to standard medications.

As it has been shown by Ansell et al., cells within the microenvironment in lymphomas express PD-L1, and with intratumorally found T cells also expressing PD-1, this discovery provides the possibility to successfully target this immune checkpoint also in malignancies of hematopoietic origin (173). In the case of pidilizumab, an international phase II study was conducted in patients with diffuse large B-cell lymphoma (DLBCL) that are undergoing autologous hematopoietic stem-cell transplantation (AHSCT). The investigators discovered that among 35 out of 66 patients with measurable disease after AHSCT, the overall response rate after pidilizumab was 51%. In addition to that, an increase of activated CD4^+^ helper and central memory T cells along with circulating CD8^+^ peripheral and central memory T cells was found, which was the first reported clinical activity of PD-1 blockage in DLBCL (143). Recently, a study examining nivolumab in relapsed or refractory Hodgkin’s lymphoma revealed a substantial therapeutic activity with an objective response rate of 87% and an acceptable safety profile in the evaluated cases (NCT01592370) (174).

**Figure 2:**
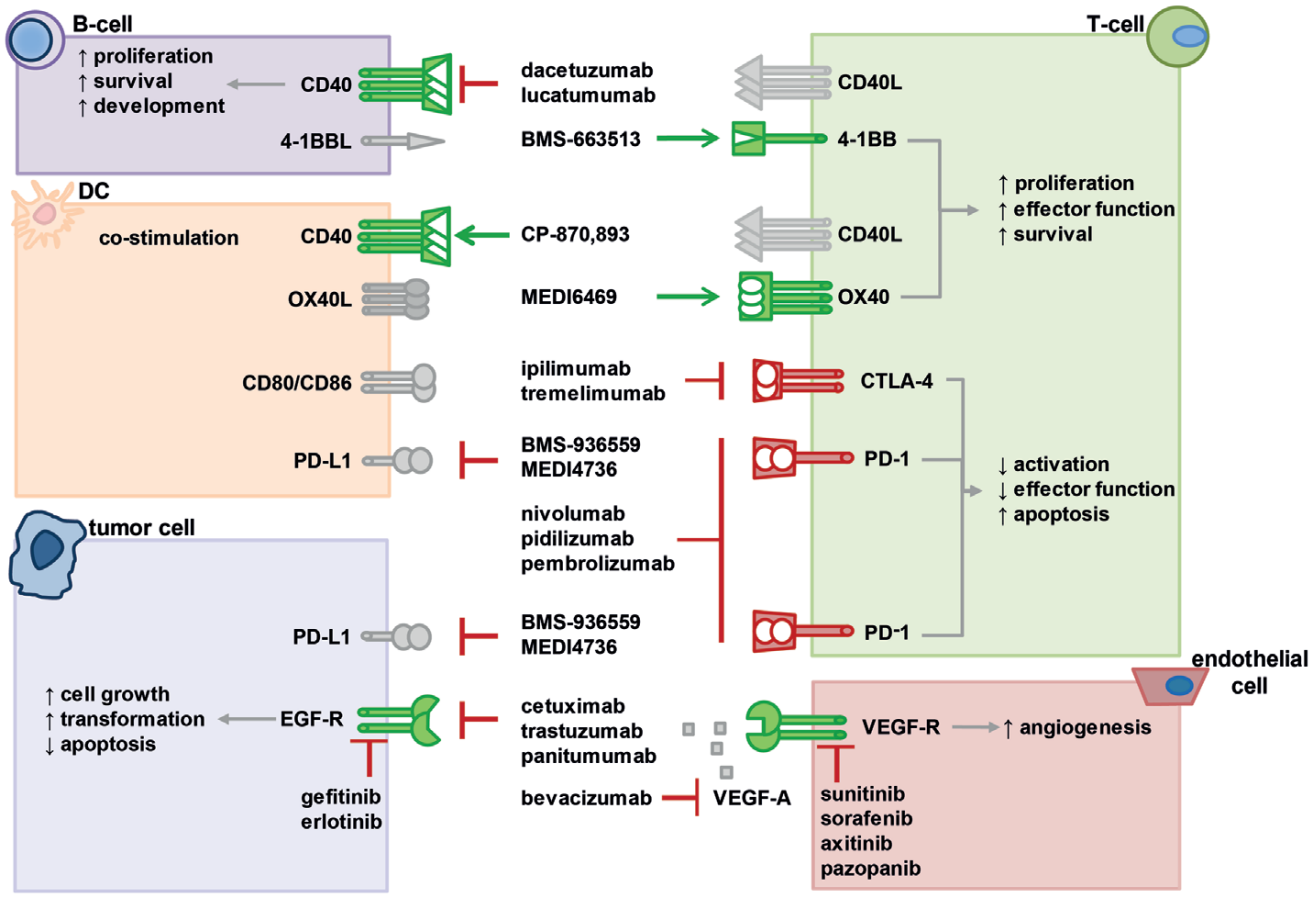
**Interactions of various co-stimulatory and inhibitory molecules regulate T-cell responses, tumor cell behavior, and vascularization**. Immunotherapies with agonistic or antagonistic monoclonal antibodies have been developed to modulate these interactions by stimulating or blocking their activity. In the figure, a selection of important molecular interactions, their most relevant cellular source (not exclusive), and examples of antagonistic (red lines) or agonistic (green arrow) monoclonal antibodies as well as inhibitors are displayed. Activating receptors are depicted in green, suppressive receptors are shown in red, ligands are gray. For further information, please refer to the main text.

In summary, PD-1 or PD-1L antagonistic mAb are able to promote a positive anti-tumor immune response in patients, while the response rate depends on the tumor entity. Thus, a combination therapy of anti-PD-1 mAb with RT could further improve the outcome and especially be an efficient strategy in the management of metastatic disease. The interactions of multiple co-stimulatory and inhibitory molecules regulating T-cell responses that can be targeted to strongly enhance radio(chemo)therapy (RCT)-induced anti-tumor immune responses are summarized in Figure 2.

## Growth Factors as Targets for Cancer Therapeutics

The activation of receptors by growth factors such as EGF, VEGF, transforming growth factor-α (TGF-α), and basic fibroblast growth factor (bFGF) triggers various cellular processes, including proliferation, differentiation, apoptosis, migration, adhesion, invasion, vascular permeability, or angiogenesis. As EGF and VEGF signaling pathways are a key feature in the development, progression, and metastatic formation in a wide range of cancer entities, they function as important targets for therapeutic Ab (175). In addition, pre-clinical models demonstrated a broad efficacy for anti-EGFR and anti-VEGF Abs alone (176–178) and in combination with RT (179). As in the case of checkpoint inhibitors, concurrent application should be most effective, since, e.g., VEGF-C expression is enhanced after irradiation (180). While many inhibitors are currently undergoing clinical evaluation, several others are already used in cancer therapy (Figure 2). Some of the FDA-approved inhibitors are anti-EGFR mAb that either work via binding the extracellular domain of EGFR (cetuximab, panitumumab, and trastuzumab) or target the intracellular EGFR domain such as the tyrosine kinase inhibitors gefitinib and erlotinib (181). FDA-approved anti-VEGFR mAb, on the other hand, inhibit angiogenesis through VEGF-A blocking [e.g., bevacizumab (BEV)] or also act as VEGFR tyrosine kinase inhibitors such as sunitinib, sorafenib, axitinib, and pazopanib (182, 183). They are approved for a variety of tumor entities, including metastatic colorectal cancer, gastric or gastro-esophageal carcinoma, renal-cell carcinoma (RCC), advanced soft tissue sarcoma, pancreatic neuroendocrine tumors (pNET), breast cancer, NSCLC, HNSCC, and glioblastoma. Several reports about the safety and efficacy of growth factor inhibitors either in the form of monotherapy or as a combinatory therapy paired with IT, CT, or RT have been released. However, targeting VEGF or EGFR alone does not always provide adequate tumor control in the clinic. In the next section, we will therefore focus on FDA-approved inhibitors in combinatory therapy settings together with RT or RCT.

### Growth factor inhibitors and RT

Patients suffering from loco-regional advanced squamous-cell carcinoma of the head and neck cancer (HNC) being treated in a phase III trial with a high-dose RT in combination with weekly cetuximab administration showed an increased loco-regional tumor control (24.4 vs. 14.9 months), along with increased median OS (49.0 vs. 29.3 months), increased median progression-free survival (PFS,17.1 vs. 12.4 months), and reduced mortality in comparison to high-dose RT monotherapy (149). A combination of erlotinib with R(C)T is also able to enhance OS as well as PFS in patients with metastatic NSCLC (154), cervical cancer (155), lung adonocarcinoma (184), or GBM (157). Tong et al. demonstrated a protective effect of a combination of sunitinib with RT on oligometastases (161). Their results were confirmed by Kao et al. who found a 75% local control and 40% distant control of oligometastases, a PFS of 34%, and an OS of 29% over a 4-year period in patients with HNC, liver, lung, kidneys, and prostate cancers that have been treated with SBRT and concomitant sunitinib therapy (162). However, a combination therapy of RT and sorafenib in comparison with standard therapy was not able to enhance the efficacy in GBM and hepatocellular carcinoma (159, 160, 185).

### Growth factor inhibitors and R(C)T

A phase II study demonstrated a near-complete or complete tumor response in 53% of patients treated with a combination of panitumumab and RCT vs. 32% of patients treated with standard RCT in patients with advanced rectal cancer with wild-type *KRAS* (186). *In vitro* studies on this matter also demonstrated an elevated level of radiosensitivity (187), while the clinical relevance of a combination of RT and adjunctive trastuzumab therapy is still under investigation. A phase II study investigating the effects of gefitinib with concomitant RCT in locally advanced HNC found a 4-year enhanced OS (74%), PFS (72%) and disease-specific survival rates (89%), respectively (153). BEV is the first approved angiogenesis inhibitor and is used in metastatic colorectal cancer, NSCLC, and breast cancer. As a result of the poor prognosis of patients with GBM and thus a need for new therapy modalities, the combination of standard RCT and anti-angiogenic antibodies such as BEV might be a promising approach in the treatment of this tumor entity. Therefore, various clinical trials dealt with this notion and revealed an extended PFS and improved life quality in newly diagnosed GBM patients that have been treated with standard RCT and BEV, whereas no change in OS was observed (147, 148, 188). BEV has also been examined in various other entities: an addition of BEV to RCT in pancreatic adenocarcinoma resulted in survival rates similar to those of standard approaches (189), in cases of nasopharyngeal carcinoma it was able to promote OS and has been linked to a delayed progression of distant metastases (145). Furthermore, an application of BEV in metastatic colorectal cancer resulted in high rates of long-term complete responses (CRs) (146). A simultaneous VEGF-A and EGFR blockade (BEV + erlotinib) in locally advanced head and neck cancer (LA-HNC) together with concurrent RCT are favorable when being compared to historical controls (158).

Taken together, all these data suggest a synergized effect of combination treatment of R(C)T with VEGF and/or EGFR inhibitors as seen in NSCLC cells reported by Kriegs et al. (190). Most of the approved mAb used in cancer IT are generally well tolerated in humans (191). Conversely, mAb application can also be associated with an increased risk of unwanted and possibly even fatal adverse effects (191–194), including cytokine release syndrome, induced autoimmunity, organ toxicity, opportunistic infections, and even cancer as a result of immune suppression. This shows how, despite the success of therapeutic Ab, their clinical efficacy greatly depends on tumor type, treatment duration, administered dose, and combination-therapy options. With this in mind, a new and promising approach in IT, the adoptive cell transfer, might be another useful therapy option to be combined with RT. Here, autologous T cells that are either tumor-specific or genetically engineered are expanded *ex vivo* before being infused back into the patient. In this article, we will focus on genetically engineered T cells only.

## Chimeric Antigen Receptors as Tool to Recognize Specific Tumor-Associated Antigens

As mentioned above, tumors are able to establish an immunosuppressive microenvironment resulting, amongst other effects, in the inhibition of an anti-tumor-specific T-cell response. This state is achieved through release of immunosuppressive cytokines, altered MHC expression, recruitment of regulatory T cells, and/or the up-regulation of immune suppressing molecules such as CTLA-4, PD-1, and PD-L1 [reviewed in Ref. (195)]. Genetically engineered T cells, possessing a cloned tumor-specific TCR or chimeric antigen receptor (CAR) and thus the ability to recognize specific TAAs, might provide a new, promising immunotherapeutic strategy for cancer treatment. CARs are constructed from an antigen-binding domain [i.e., single chain antibody variable fragment (scFv)] that is derived from the variable region (Fab-fragment) of a mAb which is linked to a transmembrane motif as well as an intracellular signaling domain of one or more co-stimulatory molecules such as CD28, Ox40, or CD137 (196).

Currently about 70 clinical trials investigating CAR T cell ITs are registered in *ClinicalTrials.gov*, with most of these studies exploring B-cell malignancies targeting CD19. One of these studies, a phase I trial of CD19-CAR T cells used in refractory B-cell malignancies, reported a CR in 70% of patients with acute lymphoblastic leukemia (B-ALL) as well as an OS at a median followup of 10 months with 51.6% at 9.7 months (197). A second study evaluating the effects of CD19-directed CAR (CTL019) T-cell therapy in relapsed or refractory ALL reported a 90% rate of complete remission (198). Along with other clinical trials (199, 200), these findings suggest a high beneficial effect of adoptive cell transfer with anti-CD19 CAR T cells in patients suffering from B-cell malignancies with manageable toxicities. These results give rise for cautious optimism in the treatment of solid tumors, including advanced Her2-positive malignancies, GBM, neuroblastoma, sarcomas, melanoma, metastatic pancreatic cancer, and metastatic breast cancer. In order to enhance anti-tumor effects of CAR T cell therapy, it can also be combined with other therapy options or the so-called bi-specific CARs recognizing two antigens that are composed of two tandem-scFv fragments separated by a linker (201). The lymphodepletive and tumoricidal effects of standard-of-care CT and RT might potentiate the expansion and function of adoptively transferred CAR T cells, as suggested by Riccione et al. (202). However, more data of combination of RT with CAR T cells are first needed to allow for definite conclusions whether this treatment induces enhanced anti-tumor responses, locally and systemically.

## Summary

A tumor is much more than just an accumulation of tumor cells. The cell death resistance of the malignant cells to anti-cancer therapies is one massive problem in the clinic. One of the challenges for researchers and clinicians is to identify treatments that will overcome or bypass the cell death resistance mechanisms established by the tumor cells, but also those of the microenvironment (68). Nowadays, the involvement of the immune system as a vital player in the recognition and eradication of malignant cells is generally accepted (203). While RT and CT are crucial for curative and palliative treatments, they do not only display cytotoxic or cytostatic effects and target the tumor directly, but are also involved in the activation of the immune system through the induction of immunogenic cell death or immunostimulatory mechanisms (29). In general, the modulation of the immune system via modifications of either tumor or immune cells with methods such as mAbs or small molecule inhibitors provides a great potential in the improvement of cancer therapies and numerous pre-clinical and clinical studies are ongoing. Even though these approaches often induce only modest and transient clinical responses in distinct malignancies, a combination with RT, and/or immunogenic CT and additional immune therapies such as vaccination might result in an improved clinical benefit. Thus, additional multi-center large-scaled randomized studies further evaluating the safety, efficacy, and clinical local and systemic outcome of monotherapy and combinatorial strategies are urgently needed. A more personalized treatment of patients through integration of predictive and prognostic biomarkers and considering individual radiosensitivity together with time and dose adaptions should be in the mind of clinicians and scientist alike. However, both have to keep in mind: it is crucial to first gain knowledge about the mechanisms and mode of action of the treatments to then be able to design multimodal therapies with respect to combinations and chronology. And if it does not work in the first try, go back to the lab and find out what can be optimized.

## Conflict of Interest Statement

The authors declare that the research was conducted in the absence of any commercial or financial relationships that could be construed as a potential conflict of interest.
